# Does Emotional Intelligence Matter in Tough Times? A Moderated Mediation Model for Explaining Health and Suicide Risk amongst Short- and Long-Term Unemployed Adults

**DOI:** 10.3390/jcm8060797

**Published:** 2019-06-05

**Authors:** Sergio Mérida-López, Natalio Extremera, Cirenia Quintana-Orts, Lourdes Rey

**Affiliations:** 1 Department of Social Psychology, Social Work, Social Anthropology and East Asian Studies, Faculty of Psychology, University of Málaga, 29071 Málaga, Spain; sergioml@uma.es; 2 Department of Social, Developmental and Educational Psychology, University of Huelva, 21004 Huelva, Spain; cquintana@uma.es; 3 Department of Personality, Assessment and Psychological Treatment, Faculty of Psychology, University of Málaga, 29071 Málaga, Spain; lrey@uma.es

**Keywords:** suicide risk, mental health, physical health, perceived stress, emotional intelligence, unemployment

## Abstract

This study contributes to knowledge on psychosomatic research by examining a moderated mediation model in which emotional intelligence (EI) is related to mental health, physical health and suicide risk through perceived stress, in samples of short-term (*n* = 364) and long-term (*n* = 594) unemployed individuals. The moderating effect of emotional intelligence on the relationships between perceived stress and mental and physical health and suicide risk was tested. The results showed that emotional intelligence was positively associated with mental and physical health and negatively associated with perceived stress and suicide risk. The proposed model only predicted mental health and suicide risk in the long-term unemployed sample. This suggests that emotional intelligence may act as a buffer against the negative impact of unemployment-related stress on mental health and suicide risk when unemployment is prolonged. Therefore, interventions targeting both the promotion of mental health and the prevention of suicide risk via the promotion of emotional abilities may consider length of unemployment.

## 1. Introduction

There is wide agreement that unemployment is one of the main socioeconomic predictors of health [1,2]. Unemployment is considered as an adverse and chronic stressor provoking a deprivation of the positive benefits typically associated with employment [3]. Unemployed individuals experience a diverse array of socio-economic stressors (e.g., financial strain, loss of work relationships, etc.), and the causal link between unemployment and higher psychological distress is well establish. Meta-analytic review has shown that unemployment is associated with reduced emotional wellbeing and physical complaints [4]. This association between unemployment and decreased emotional wellbeing contributes to an explanation of the fact that unemployed individuals have worse psychological health than their employed counterparts [5]. 

A number of studies have provided data on the impact of being unemployed on a wide range of health outcomes, including distress, physical complaints, mental disorders, and suicidality [1,6,7]. Suicide risk has been underscored as a critical ill-health indicator among the unemployed. For instance, it has been demonstrated that being unemployed is associated with an increased risk of suicide, irrespective of prior mental health status [8]. In sum, a great deal of attention has been paid to the impact of a stressor such as unemployment on individuals’ health and wellbeing, resulting in a growing body of evidence in the fields of health psychology and psychosomatic medicine [1,9]. However, less is known about the psychological resources that may reduce the impact of unemployment on an individual’s health. 

Literature reviews carried out in recent decades have suggested several pathways linking the experience of being unemployed with poorer health outcomes, with stress having a strong influence on disease and suicidality [10,11,12]. Korpi (2001) argued that stress may explain the deleterious influence of unemployment on physical health or suicide [9]. Moreover, meta-analytic studies have shown stress appraisal to be a key dimension in explaining decreased psychological health amongst the unemployed [4]. Stressful situations such as unemployment might impact negatively on individuals over time, causing them health problems or to suffer other emotional adverse reactions (i.e., suicidal ideation). In summary, reviews of stress and coping literature have widely shown the role of perceived stress as a contributor to aspects of psychological maladjustment across different unemployment samples. Likewise, stress appraisal is regarded as an underlying mechanism that might explain the adverse psychosocial impact of unemployment on mental and physical health and suicide risk [9,11]. 

In a meta-analytic review, Paul and Moser (2009) concluded that unemployment had a stronger negative effect on individuals’ wellbeing and mental health when it persisted [5]. Moreover, Milner et al. (2014) showed a positive association between exposure to unemployment and suicide risk [8]. These findings may be explained in terms of coping strategies used by short- and long-term unemployed individuals [13]. In fact, nonproductive coping was found to explain the association between length of unemployment and somatic complaints [13]. Elsby and colleagues (2010) showed that length of unemployment may be critical, as employers tend to hire short-term rather than long-term unemployed individuals [14]. More recently, it has been shown that job-finding becomes harder after the first 12 months of unemployment [15]. These alarming findings have led to an examination of the differences between short- and long-term unemployment amongst adults regarding their psychological wellbeing or negative affective experience [16,17]. Despite the emerging consensus that length of unemployment is a key sociodemographic variable, no studies to date have examined the role of psychological resources on stress and health outcomes regarding different groups of unemployed individuals.

### 1.1. Emotional Intelligence as a Psychological Resource Explaining Stress and Health Outcomes in Unemployed Individuals

Wanberg (2012) argued that there is need for evidence on the processes through which individual characteristics influence health status so that more effective interventions can be developed for people who are unemployed [1]. Psychosomatic research increasingly focuses on the psychological resources that individuals have at their disposal to prevent illness when coping with deleterious and chronic psychosocial stressors such as unemployment [18,19]. It has been reported that people display a wide repertoire of adaptations to the stress associated with unemployment, which affects health and suicide risk [4,13]. Hence, efforts have been directed at broadening models of health and wellbeing in the unemployed, and there is now more interest in examining the protective role that psychological resources play during periods of unemployment [1,20]. It has been suggested that emotional intelligence (EI), a construct that captures individuals’ abilities to deal with affective information, is a core psychological resource and predictor of stress and health outcomes in a variety of settings, including unemployment [21,22]. 

The ability perspective on EI defines this construct as a set of cognitive-emotional skills for processing emotional information in order to promote emotional and intellectual growth [23]. Recent reviews have shown that the EI construct predicts physical and mental health indicators [21,22], as well as suicide risk [24]. Beyond the direct influences of EI on health and suicide risk, there is consistent evidence showing that EI significantly influences stress responses which, in turn, can impact health and suicide risk. EI is negatively associated with the cortisol response to induced stress, even after controlling for the variance due to personality traits [25]. Moreover, EI influences the extent to which laboratory stressors are perceived as threatening [26]. Similarly, unemployed adults’ EI has been suggested as a psychological resource associated with lower levels of stress symptoms [27] and mental health disorders [28]. Second, it is plausible to expect that EI may buffer the effects of stress on general health and suicide risk among the unemployed. Accordingly, an experimental study with unemployed adults found that promotion of EI was associated with decreases in stress and mental health [29]. Additionally, EI has been seen as a buffer against the effects of unhappiness and dissatisfaction with life and suicide risk in unemployed adults [19]. In sum, there are several theoretical and empirical reasons to expect not only a significant relationship between EI and health outcomes (i.e., general mental and physical health and suicide risk) through stress, but also to expect EI to buffer the associations between stress and health outcomes. 

### 1.2. Purpose, Objectives and Hypotheses of the Research

**Figure 1:**
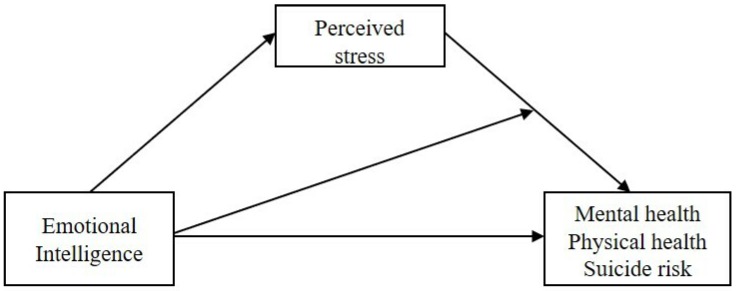
Proposed model of the relationships between emotional intelligence (EI), perceived stress and mental health, physical health, and suicide risk.

Although EI is currently recognized as protecting against ill-health and suicide, there is limited evidence on EI as a psychological resource in the context of unemployment. The aim of this research, therefore, was to contribute to psychosomatic research in three ways. Firstly, in an attempt to plug the gap in evidence on the role of EI in enhancing health and reducing suicide risk in the unemployed, we proposed a moderated mediation model examining (1) whether the relationship between EI and health and suicide risk is mediated by perceived stress, and (2) whether an indirect association between EI and health and suicide risk via perceived stress is dependent on EI levels. The proposed model is displayed in Figure 1. Secondly, we attempted to validate our proposed model in two samples, differing with respect to length of unemployment (i.e., short- and long-term unemployment) to provide evidence on generalizability and new information about EI as a psychological resource for the unemployed. Finally, we hoped that the novel findings of our proposed model would provide evidence that might be used to develop interventions to reduce the deleterious impact of unemployment on health.

Based on the above-mentioned literature, we proposed that EI has an indirect relationship with general mental health (H1A), general physical health (H2A), and suicide risk (H3A), which is mediated by perceived stress (mediation hypotheses). Second, we expect the indirect effect of EI on health outcomes through perceived stress to be dependent on EI levels. In particular, we predict that EI would moderate the relationship between stress and general mental health (H1B), general physical health (H2B), and suicide risk (H3B).

## 2. Materials and Methods

### 2.1. Participants

A total of 958 unemployed adults (54.6% female) participated in our research. As we wanted to compare short- and long-term unemployment, we defined long-term unemployment as unemployment lasting 12 months or more. This criterion for long-term unemployment was followed considering data from the Spanish Health Survey [7]. Likewise, prior research has considered this same criterion when comparing samples of short- and long-term unemployed adults [16,30,31]. Those participants reporting lengths of unemployment between less than a month and less than 12 months were classed as short-term unemployed (sample 1), whereas those who had been unemployed for 12 months or longer were classed as long-term unemployed (sample 2). 

Sample 1 comprised 364 short-term unemployed adults (56.3% female) with a mean age of 32.57 years (SD = 10.69). Most reported their marital status as single (56.6%) or married (28.3%). Most reported their educational level as primary studies (35.2%) or secondary studies (32.2%). The mean length of unemployment was 4.23 months (SD = 2.90). 

Sample 2 comprised 594 long-term unemployed adults (53.5% female) with a mean age of 37.40 years. Most reported their marital status as single (43.4%) or married (40.7%). The most commonly reported educational levels were primary (37.2%) and secondary (37.9%) studies. The average length of unemployment was 34.55 months (SD = 36.30).

### 2.2. Design and Procedure

A cross-sectional design was used to examine the proposed moderated mediation model (see Figure 1). To enhance the generalizability of the findings the model, two different samples were tested regarding length of unemployment (i.e., short- and long-term unemployed adults). 

A snowball sampling technique was used to recruit respondents [32]. Potential participants were approached and asked to voluntarily take part in a study on “unemployment, health, and wellbeing” with the help of university students. Data collection took place in different national employment agencies in southern Spain. Students returned completed questionnaires to the research staff for data processing. As in previous works, this sampling technique was chosen because of its practical advantages in field studies with sensitive samples such as unemployed adults [33,34]. It has been argued that this technique may constitute a suitable alternative to data collection through unemployment services [33]. Nonetheless, several recommendations were addressed to avoid the potential limitations of this technique [32]. First, inclusion and exclusion criteria were applied. The inclusion criteria were being aged above 18 years old, unemployed at the time of questionnaire completion and willing to participate in the research. Conversely, exclusion criteria were being unemployed but not actively job searching (e.g., medical condition) or illiteracy in Spanish. Second, basic sociodemographic data were collected (i.e., age, gender, educational level, marital status, and length of unemployment). Finally, the self-administered questionnaires were in paper-and-pencil format, and included precise written instructions in order to avoid the traditional drawbacks of snowball sampling [32]. 

No financial compensation was offered to the unemployed adults for their participation. Participants were aware that by filling in the surveys they were consenting to the use of their data in this research. As in prior research involving psychological health and suicide risk [35], it was made clear that participants could stop at any point if they became distressed whilst filling in the questionnaire. Participants took, on average, 20 min to complete the questionnaire. The study was conducted in accordance with the Declaration of Helsinki, and the protocol was approved by the Research Ethics Committee of the University of Málaga (66-2018-H).

### 2.3. Measures

The questionnaire collected data on several sociodemographic variables relevant to the main study variables: gender, age, educational level, marital status, and length of unemployment. 

Emotional intelligence was measured using the Spanish adaptation [36] of the Wong and Law Emotional Intelligence Scale [37]. The instrument consists of 16 items that are on a 7-point Likert scale (1 = “totally disagree” to 7 = “totally agree”). The scale assesses the subscales of appraisal of one’s own emotions, appraisal of others’ emotions, use of emotion and regulation of emotion (e.g., “I always encourage myself to try my best”). However, we used the overall score in our analyses as we were interested in the global EI score [19,37]. The instrument has shown adequate reliability in previous studies [19,27]. 

Perceived stress was assessed with the short, four-item version of the Perceived Stress Scale [38], in a validated Spanish version [39]. Respondents used a five-point Likert scale ranging from 0 (“never”) to 4 (“very often”) to rate the frequency of experiencing stress during the past month (e.g., “In the last month, how often have you been upset because of something that happened unexpectedly?”). The scale has shown adequate psychometric properties in Spanish samples [40]. 

General mental and physical health were assessed using the Spanish adaptation [41] of the 12-item, short-form health survey [42]. The scale yields two overall scores: the Mental Composite Summary (MCS) and the Physical Composite Summary (PCS), with higher scores indicating a better general state of health. The instrument has shown adequate reliability [34]. 

Suicide risk was assessed using the Suicidal Behaviors Questionnaire-Revised (SBQ-R) [43]. The four items of the instrument are scored on a Likert scale ranging from 0 or 1 (“never”) to 5 (“very often”) or 6 (“very likely”), such that higher scores indicate greater suicidal behavior. The instrument assesses lifetime suicidal ideation and attempt, frequency of suicidal ideation in the past year, communication of suicidal behavior, and self-reported likelihood of future suicidal behavior. The Spanish version has shown adequate reliability [19]. 

## 3. Results

### 3.1. Descriptive Analyses and Correlations

Table 1 shows the means, standard deviations, reliabilities and correlations of all the study variables in samples 1 and 2. In order to test the overall differences between samples regarding the mediator and dependent variables, independent *t*-tests were used. In comparison with short-term unemployed adults, long-term unemployed adults reported higher perceived stress (*t*(956) = −2.25, *p* < 0.05; *d* = 0.15), lower MCS (*t*(956) = 2.15, *p* < 0.05; *d* = 0.14), and lower PCS (*t*(872.10) = 2.30, *p* < 0.05; *d* = 0.15). There were no significant differences regarding suicide risk (*t*(956) = −0.04, *p* = 0.97). 

Bivariate correlation analyses showed similar results in both samples. In short, there were negative correlations between EI and perceived stress and between EI and suicide risk. Conversely, EI was positively associated with both MCS and PCS. Moreover, perceived stress was positively related to suicide risk and negatively linked to both MCS and PCS. Lastly, MCS and PCS were negatively associated with suicide risk.

### 3.2. Testing of Control Variables

Preliminary analyses were conducted to examine potential cofounding effects of sociodemographic variables such as gender, age, and length of unemployment on outcomes. In sample 1 there were gender differences in MCS (*t* = 3.41, *p* < 0.01) and PCS (*t* = 2.13, *p* < 0.05), with men’s scores higher than women’s scores. Suicide risk was similar for men and women (*t* = −1.34, *p* = 0.17). There were negative associations between age and MCS (*r* = −0.11, *p* < 0.05) and between age and PCS (*r* = −0.17, *p* < 0.01). Age and suicide risk were not associated (*r* = 0.02, *p* = 0.64). Length of unemployment was not associated with MCS (*r* = −0.03, *p* = 0.52), PCS (*r* = −0.04, *p* = 0.45) or suicide risk (*r* = −0.01, *p* = 0.92). 

In sample 2 there were no gender differences in MCS (*t* = 0.95, *p* = 0.34), PCS (*t* = 1.04, *p* = 0.30), or suicide risk (*t* = 1.26, *p* = 0.21). Although age was negatively associated with PCS (*r* = −0.24, *p* < 0.01), it was not associated with MCS (*r* = 0.01, *p* = 0.98) or suicide risk (*r* = −0.06, *p* = 0.17). Length of unemployment was not related to MCS (*r* = 0.08, *p* = 0.06) or suicide risk (*r* = −0.07, *p* = 0.09), but it was negatively associated with PCS (*r* = −0.08, *p* < 0.05). On this basis, gender and age were included in subsequent analyses as covariates.

### 3.3. Conditional Process Analyses and Hypotheses Testing

In both samples we tested (a) whether EI had an indirect relationship with MCS, PCS, or suicide risk mediated by perceived stress (mediation hypotheses: H1A, H2A, and H3A), and (b) whether the indirect relationship between EI and MCS, PCS, or suicide risk mediated by perceived stress was dependent on the level of EI (moderation hypotheses: H1B, H2B, and H3B). For this purpose, various moderated mediation models were estimated using bootstrapping techniques with the PROCESS macro for SPSS (Model 74). Since PROCESS provides similar results to structural equation modeling, we regarded it as a convenient tool for carrying out conditional process analyses [44]. Consistent with the proposed model (see Figure 1), overall EI was entered as the predictor variable (X); MCS, PCS, and suicide risk scores were the dependent variables (Y); and perceived stress was the mediator (M).

Given that we conducted multiple regression by means of the PROCESS macro and this technique generates two multiple regression models for each dependent variable, familywise error was a potential issue. In line with prior research [45], a Bonferroni correction was used where the total number of analyses (n) was six for each sample (α = 0.05/6 = 0.008). Thus, the significance of the obtained coefficients in the moderated mediation analyses was tested considering a Bonferroni-corrected significance threshold of *p* = 0.008. Gender and age were statistical controls. In accordance with guidelines we used 5000 bootstrap resamples and calculated 95% CI in all analyses. 

#### 3.3.1. General Mental Health

The main results of the moderated mediation models for sample 1, comprised of short-term unemployed adults, are summarized in Table 2. First, the mediator variable model showed overall EI was negatively related to perceived stress (*b* = −0.26, *p* < 0.008, 95% CI = −0.33, −0.19). In the dependent variable model, perceived stress predicted the MCS (*b* = −6.45, *p* < 0.008, 95% CI = −7.93, −4.96). Thus, it was supported that short-term unemployed adults’ EI influence on MCS is mediated by perceived stress (H1A). Second, although EI predicted the MCS (*b* = 1.43, *p* < 0.008, 95% CI = 0.40, 2.45), there was no significant interaction between EI and perceived stress (*b* = 1.14, *p* = 0.089), and so it was rejected that EI moderates the relationship between perceived stress and MCS (H1B). 

Results of the analyses of sample 2, consisting of long-term unemployed adults, are displayed in Table 3. First, EI was negatively linked to perceived stress (*b* = −0.28, *p* < 0.008, 95% CI = −0.33, −0.23), which in turn predicted the MCS (*b* = −6.81, *p* < 0.008, 95% CI = −7.95, −5.67). Thus, it was found that perceived stress mediates the relationship between EI and the MCS (H1A). Second, MCS was predicted by EI (*b* = 1.85, *p* < 0.008, 95% CI = 1.04, 2.67), and the interaction between EI and perceived stress was significant (*b* = 1.37, *p* < 0.008, 95% CI = 0.46, 2.27). It was supported that the effect of perceived stress on MCS is moderated by EI levels (H1B). We followed the procedure by Hayes [44] to determine the values of EI at which the conditional indirect effects of perceived stress on MCS were significant. The moderated mediation index was significant (coeff. = −0.38, 95% CI = −0.61, −0.17), indicating that the conditional indirect effects estimated at low and high levels of the moderator were different from each other [46].

**Figure 2:**
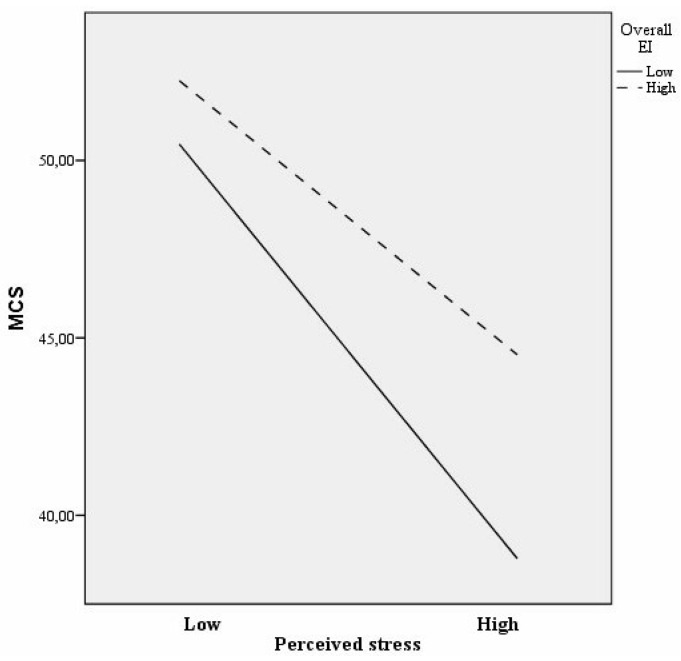
Interaction between EI and perceived stress with regard to MCS in the long-term unemployed sample.

Figure 2 illustrates the interaction between EI and perceived stress with respect to MCS in the long-term unemployed sample. Perceived stress and MCS were negatively associated at low EI levels (*b* = −8.20, *t*(588) = −10.68, *p* < 0.008), but the association was weaker at high EI levels (*b* = −5.42, *t*(484) = −7.46, *p* < 0.008). Post hoc analyses showed that the slopes of the two lines were different (*t* = 2.63, *p* < 0.008). In sum, results indicated that EI predicted higher levels of MCS through decreased stress and, furthermore, may buffer the negative association between perceived stress and worsened mental health among the long-term unemployed individuals.

#### 3.3.2. General Physical Health

Regarding sample 1, overall EI was negatively related to perceived stress (*b* = −0.26, *p* < 0.008, 95% CI = −0.33, −0.19). However, PCS was not significantly predicted by perceived stress after the Bonferroni correction (*b* = −1.38, *p* = 0.018, 95% CI = −2.52, −0.23), so H2A was rejected. EI did not significantly predict the PCS after applying the Bonferroni correction, (*b* = 0.93, *p* = 0.021) and the interaction between EI and perceived stress was insignificant (*b* = 0.19, *p* = 0.71), thereby rejecting H2B. 

Regarding sample 2, EI predicted perceived stress (*b* = −0.28, *p* < 0.008, 95% CI = −0.33, −0.23), but perceived stress did not predict the PCS (*b* = −0.65, *p* = 0.20), thereby rejecting H2A. Finally, there was no significant interaction between EI and perceived stress (*b* = −0.24, *p* = 0.56), so H2B was rejected. In sum, neither the mediation nor the moderation hypotheses regarding general physical health as outcome were supported in any sample.

#### 3.3.3. Suicide Risk

In sample 1, EI was related to perceived stress (*b* = −0.26, *p* < 0.008, 95% CI = −0.33, −0.19). Suicide risk was predicted by perceived stress (*b* = 0.62, *p* < 0.008, 95% CI = 0.27, 0.98), thereby supporting H3A. Although EI significantly predicted suicide risk (*b* = −0.39, *p* < 0.008, 95% CI = −0.64, −0.15), the interaction between EI and perceived stress was insignificant after applying the Bonferroni correction (*b* = −0.32, *p* = 0.048). Thus, H3B was rejected. 

In sample 2, EI was related to perceived stress (*b* = −0.28, *p* < 0.008, 95% CI = −0.33, −0.23), which was related to suicide risk (*b* = 0.71, *p* < 0.008, 95% CI = 0.43, 0.99). Thus, H3A was supported. Although suicide risk was not significantly predicted by EI after applying the Bonferroni correction (*b* = −0.26, *p* = 0.013), the interaction between EI and perceived stress was significant and so H3B was confirmed (*b* = −0.61, *p* < 0.008, 95% CI = −0.84, −0.38). The moderated mediation index supported this result (coeff. = 0.17, 95% CI = 0.07, 0.29). 

**Figure 3:**
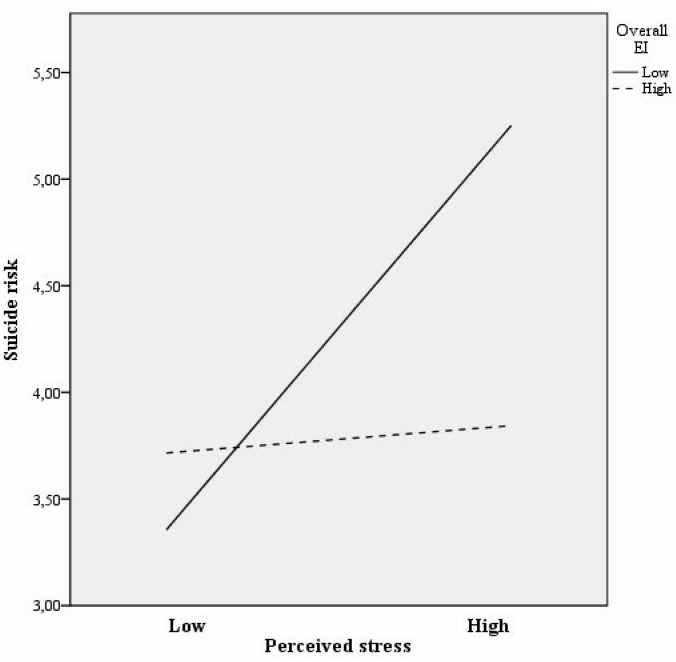
Interaction between EI and perceived stress with regard to suicide risk in the long-term unemployed sample.

Figure 3 illustrates the interaction between EI and perceived stress with suicide risk as a dependent variable in the sample 2. At low levels of EI there was a significant association between perceived stress and suicide risk (*b* = 1.33, t(588) = 6.94, *p* < 0.008), but it became insignificant at high EI levels (*b* = 0.09, *t*(588) = 0.50, *p* = 0.62). Finally, post hoc analyses showed that the slopes of the two lines were different (*t* = 4.71, *p* < 0.008). 

## 4. Discussion

The aim of this study was to contribute to the psychosomatic research literature by examining perceived stress as a potential mediator of the relationships between EI and general health and suicide ideation in two samples of unemployed adults. Our findings corroborated that the significant relationship between EI, general mental health (H1A), and suicide risk (H3A) is mediated by perceived stress in both short- and long-term unemployed adults. This finding was in line with previous studies relating EI, stress, and health outcomes. Likewise, our results suggest a need to consider EI as a potential psychological resource that might mitigate stress among the unemployed [21,29]. However, we failed to provide evidence of the role of perceived stress as a mediator in the relationship between EI and general physical health among the unemployed (H2A). Wanberg (2012) has argued that the mechanisms relating unemployment with a worsened physical health remain unclear in comparison with current evidence on the effects of unemployment on psychological wellbeing and suicide. Likewise, reviews of the literature on EI and physical health show that the relationship between EI and physical health outcomes are complex. Undoubtedly, further studies are needed to clarify the mechanisms linking unemployed adults’ EI to their physical health [21,22]. 

Results have shown that EI moderates the relationship between perceived stress and general mental health (H1B) and suicide risk (H3B) in long-term unemployed adults. In particular, individuals reporting high perceived stress and low EI reported both the lowest scores in MCS and the highest scores in suicide risk. These results can be understood in light of the coping strategies that individuals use regarding their levels of EI [21]. Low-EI unemployed adults are more likely to engage in maladaptive strategies [34] which, in turn, may contribute to maintaining levels of negative affect [13]. In sum, these findings suggest EI may act as a psychological resource, potentially explaining a more adaptive coping with unemployment-related stress [13] and, in turn, facilitating a lower impact of stress on ill-health and risk of suicide [19,29]. 

Contrary to our expectations, results did not support the potential moderating role of EI in the association between perceived stress and unemployed adults’ general physical health (H2B). This insignificant finding might be explained in terms of the complex relationship between EI, stress, and physical health [22], and future works could beneficially assess more specific aspects of physical health such as physical and somatic complaints [13,29]. Likewise, one promising avenue would be to investigate healthy lifestyle behaviors associated with emotional abilities among unemployed adults [47].

### 4.1. Limitations

This study had several limitations that should be considered when interpreting our results. First, although our model was grounded in theoretical and empirical evidence relating EI, stress, and health outcomes [8,21,22], the cross-sectional design of the study limited the interpretation of our associations, as it is not possible to determine causal associations. These preliminary findings would be strengthened by means of longitudinal designs [4,8]. Given that the relationships amongst employment status, mental disorders, and suicide are complex, future works are advised to overcome limitations of this research by considering competitive models with direct and reciprocal associations amongst variables [8]. Following the systematic review by Pompili et al. (2015), which showed that physical health issues could be risk factors for suicide, there is a need for longitudinal research to determine whether risk of suicide as a consequence of mental disorders and physical ill-health can be attenuated by EI [34]. This future line would constitute a promising avenue for applying current results. 

Secondly, the relatively low internal consistency of the perceived stress scale that was used should be acknowledged. This fact has been explained in terms of the number of items of this shorter version in comparison with the original one [48]. Although the short version of the scale may be useful in settings in which assessment time is limited [38], future studies should replicate these results using either a longer version [38] or complementary measures such as stress symptoms [27].

Thirdly, the use of self-report measures may be associated with common method biases. Future studies using objective measures of EI (e.g., performance-based tests), stress (e.g., biochemical indices), and health (e.g., health symptom checklists) would valuably reduce this issue, thereby increasing the generalizability of our findings [25]. 

Finally, there was a limitation regarding the snowball sampling method used. Although we followed recommendations regarding the use of this technique to avoid potential issues [32,34], data might have been more biased toward more cooperative participants who were willing to participate in our research, which may be a risk for the generalizability of our findings. Thus, future research is strongly advised to use random sampling procedures together with a more heterogeneous sample selection in terms of geographic characteristics.

### 4.2. Implications 

Despite the aforementioned limitations, this research has provided new evidence for the proposed moderated mediation model, and suggested further avenues for reducing the impact of unemployment on health and suicide risk amongst adults. Our proposed model did not account for significant outcome variance in the short-term unemployed sample, whereas in the long-term unemployed sample it accounted for a statistically significant proportion of variance in mental health and suicide risk. Prior research has suggested that under conditions of increasing distress, individuals’ efforts at mood repair may help to account for reported frequency of illnesses [49]. Additionally, a meta-analysis concluded that length of unemployment moderates the deleterious impact of unemployment on health and wellbeing [5]. These findings suggest that the contribution of EI to variance in health-related outcomes in unemployed people depends on length of unemployment—that is, EI may be a psychological resource that helps attenuate the suicide risk associated with long-term unemployment and protects the mental health of long-term unemployed adults who may be exposed to greater stress than those who have only been unemployed for a short time [49]. 

Consistent with the notion that EI might be particularly useful for individuals experiencing high stress related to long-term unemployment [49], our findings extend current knowledge about the psychosomatic effects of unemployment by providing empirical evidence on the extent to which variance in EI accounts for variance in illness and suicide risk indicators, thereby providing some insight into the practical implications of this research. First, the possibility of using self-report ability-based EI tests to screen for profiles that predict increased risks of stress, mental ill-health, and suicide risk could be examined by researchers and practitioners. As experience of unemployment negatively impacts health [4], an implication of these and previous findings would be to determine which long-term unemployed adults are at a greater risk of suicide [19]. 

Second, our findings suggest practical implications for improving mental health prevention efforts during unemployment. In fact, unemployed adults visiting local offices for employment could be offered EI training. Prevention strategies such as emotional ability modules, which are designed to train unemployed individuals how to identify emotions, use feelings to generate alternative solutions. These strategies could include training on how emotions influence all workplace decision making, as well as how to understand the causes and consequences of negative mood states over time, and potential strategies for managing unhelpful emotions that might reduce the likelihood of negative mental health or suicidal outcomes. Although EI training alone will undoubtedly not be sufficient to eliminate the adverse consequences of long-term unemployment, current evidence shows promising results on the positive effects of EI on health outcomes. For example, Hodzic and colleagues (2015) reported that unemployed adults who received an EI intervention showed fewer somatic complaints, lower perceived stress, and better mental health and vigor six months after the intervention when compared with the control group [29]. Our results suggest that EI training could foster general health via attenuating the effects of perceived stress on mental ill-health and suicide risk. Thus, EI training focusing on the use of adaptive coping strategies during the unemployment situation may be a promising approach to mitigate stress levels and reduce potential mental and physical health issues found in long-term unemployment [4,13]. In sum, our findings suggest that EI training programs should be tailored to the needs of different groups of unemployed individuals.

## 5. Conclusions

Our study calls for further research examining the potential role of EI and perceived stress in mental health outcomes, and it also highlights a need for better understanding how EI may place some at greater protection for maladjustment, especially among long-term unemployed people. We hope that our findings will help future scholars to develop and examine more comprehensive models that consider psychological resources such as EI in the experience of unemployment. The present findings suggest not only using low-EI as a screening tool for predicting high stress and subsequent maladjustment, but also to consider these results in designing effective approaches that prevent the effects of stress on mental health and suicide risk, especially among long-term unemployed people.

## Figures and Tables

**Table 1:** Descriptive statistics and bivariate correlations in samples 1 and 2.

	M	SD	α	1	2	3	4	5
**Sample 1** (*n* = 364)								
1. Emotional Intelligence	5.06	0.96	0.92	-				
2. Perceived stress	1.59	0.66	0.66	−0.38 **	-			
3. MCS	47.64	10.19	0.76	0.31 **	−0.47 **	-		
4. PCS	52.02	6.95	0.67	0.19 **	−0.20 **	0.11 *	-	
5. Suicide risk	4.21	2.18	0.77	−0.26 **	0.25 **	−0.35 **	−0.19 **	-
**Sample 2** (*n* = 594)								
1. Emotional Intelligence	5.00	1.02	0.92	-				
2. Perceived stress	1.70	0.71	0.68	−0.40 **	-			
3. MCS	46.11	10.95	0.76	0.36 **	−0.51 **	-		
4. PCS	50.87	8.36	0.76	0.12 **	−0.12 **	0.04	-	
5. Suicide risk	4.22	2.44	0.80	−0.24 **	0.25 **	−0.36 **	−0.11 **	-

Abbreviation: M: mean; SD: standard deviation; MCS: mental composite summary; PCS: physical composite summary. * *p* < 0.05, ** *p* < 0.01.

**Table 2:** Tested dependent variable models with mental health composite, physical health composite, and suicide risk as outcomes for short-term unemployed.

	B	SE b	*R* ^2^	95% CI
**Mental Composite Summary**			0.28 **	
Constant	54.13 **	1.98		50.24 to 58.02
Gender	−3.16 **	0.93		−4.98 to −1.34
Age	−0.04	0.04		−0.13 to 0.05
EI	1.43 **	0.52		0.40 to 2.45
Perceived stress	−6.45 **	0.76		−7.93 to −4.96
EI x perceived stress	1.14	0.67		−0.17 to 2.45
**Physical Composite Summary**			0.08 **	
Constant	56.98 **	1.52		53.99 to 59.97
Gender	−1.18	0.71		−2.58 to 0.22
Age	−0.09	0.03		−0.16 to −0.03
EI	0.93	0.40		0.14 to 1.72
Perceived stress	−1.38	0.58		−2.52 to −0.23
EI x perceived stress	0.19	0.51		−0.82 to 1.20
**Suicide risk**			0.11 **	
Constant	3.82 **	0.47		2.90 to 4.75
Gender	0.24	0.22		−0.19 to 0.67
Age	−0.00	0.01		−0.02 to 0.02
EI	−0.39 **	0.12		−0.64 to −0.15
Perceived stress	0.62 **	0.18		0.27 to 0.98
EI x perceived stress	−0.32	0.16		−0.63 to −0.00

Abbreviation: EI: emotional intelligence; B: Beta; SE b: Standard error; *R*^2^: *R*-squared; 95% CI = 95% Confidence Intervals; ** *p* < 0.008 (after Bonferroni correction).

**Table 3:** Tested dependent variable models with mental health composite, physical health composite and suicide risk as outcomes for long-term unemployment.

	B	SE b	*R* ^2^	95% CI
**Mental Composite Summary**			0.31 **	
Constant	48.45 **	1.70		45.10 to 51.80
Gender	−2.09 **	0.76		−3.59 to −0.60
Age	0.03	0.03		−0.03 to 0.10
EI	1.85 **	0.42		1.04 to 2.67
Perceived stress	−6.81 **	0.58		−7.95 to −5.67
EI x perceived stress	1.37 **	0.46		0.46 to 2.27
**Physical Composite Summary**			0.08 **	
Constant	58.47 **	1.50		55.53 to 61.41
Gender	−0.81	0.67		−2.12 to 0.51
Age	−0.17 **	0.03		−0.23 to −0.12
EI	0.98 **	0.37		0.27 to 1.70
Perceived stress	−0.65	0.51		−1.66 to 0.35
EI x perceived stress	−0.24	0.41		−1.04 to 0.56
**Suicide risk**			0.13 **	
Constant	4.71 **	0.43		3.88 to 5.55
Gender	−0.05	0.19		−0.43 to 0.32
Age	−0.02	0.01		−0.03 to 0.00
EI	−0.26	0.10		−0.46 to −0.05
Perceived stress	0.71 **	0.15		0.43 to 0.99
EI x perceived stress	−0.61 **	0.12		−0.84 to −0.38

Abbreviation: EI: emotional intelligence; B: Beta; SE b: Standard error; *R*^2^: *R*-squared; 95% CI: 95% Confidence Intervals; ** *p* < 0.008 (after Bonferroni correction).

## References

[1] Wanberg C.R. (2012). The Individual Experience of Unemployment. Annu. Rev. Psychol..

[2] Huppert F.A. (2009). Psychological Well-being: Evidence Regarding its Causes and Consequences. Appl. Psychol. Heal. Well-Being.

[3] Jahoda M. (1982). Employment and Unemployment: A Social-Psychological Analysis.

[4] McKee-Ryan F.M., Song Z., Wanberg C.R., Kinicki A.J. (2005). Psychological and physical well-being during unemployment: A meta-analytic study. J. Appl. Psychol..

[5] Paul K.I., Moser K. (2009). Unemployment impairs mental health: Meta-analyses. J. Vocat. Behav..

[6] Béland F., Birch S., Stoddart G. (2002). Unemployment and health: contextual-level influences on the production of health in populations. Soc. Sci. Med..

[7] Farré L., Fasani F., Mueller H. (2018). Feeling useless: The effect of unemployment on mental health in the Great Recession. IZA J. Labor Econ..

[8] Milner A., Page A., LaMontagne A.D. (2014). Cause and effect in studies on unemployment, mental health and suicide: A meta-analytic and conceptual review. Psychol. Med..

[9] Korpi T. (2001). Accumulating disadvantage: Longitudinal analyses of unemployment and physical health in representative samples of the Swedish population. Eur. Sociol. Rev..

[10] Cohen S., Janicki-Deverts D., Miller G.E. (2007). Psychological Stress and Disease. JAMA.

[11] Schneiderman N., Ironson G., Siegel S.D. (2005). Stress and Health: Psychological, Behavioral, and Biological Determinants. Annu. Rev. Clin. Psychol..

[12] Turecki G., Brent D.A. (2016). Suicide and suicidal behaviour. Lancet.

[13] Langens T.A., Mose E. (2006). Coping with unemployment: Relationships between duration of unemployment, coping styles, and subjective well-being. J. Appl. Biobehav. Res..

[14] Elsby M.W., Hobijn B., Sahin A. (2010). The labor market in the Great Recession. Brookings Pap. Econ. Act..

[15] Bentolila S., García-Pérez J.I., Jansen M. (2017). Are the Spanish long-term unemployed unemployable?. SERIEs.

[16] Griep Y., Baillien E., Vleugels W., Rothmann S., De Witte H. (2014). Do they adapt or react? A comparison of the adaptation model and the stress reaction model among South African unemployed. Econ. Ind. Democr..

[17] De Witte H., Hooge J., Vanbelle E. (2010). Do the long-term unemployed adapt to unemployment?. Rom. J. Pplied Psychol..

[18] Creed P.A., Lehmann K., Hood M. (2009). The relationship between core self-evaluations, employment commitment and well-being in the unemployed. Pers. Individ. Dif..

[19] Extremera N., Rey L. (2016). Attenuating the Negative Impact of Unemployment: The Interactive Effects of Perceived Emotional Intelligence and Well-Being on Suicide Risk. PLoS ONE.

[20] McKee-Ryan F.M., Kinicki A.J. (2002). The personal meaning of job loss: Appraisal and coping at the facet level. Int. Rev. Ind. Organ. Psychol..

[21] Matthews G., Zeidner M., Roberts R.D., Cooper C.L., Quick J.C. (2017). Emotional Intelligence, Health, and Stress. The Handbook of Stress and Health: A Guide to Research and Practice.

[22] Martins A., Ramalho N., Morin E. (2010). A comprehensive meta-analysis of the relationship between Emotional Intelligence and health. Pers. Individ. Dif..

[23] Mayer J.D., Salovey P., Salovey P., Sluyter D. (1997). What is emotional intelligence?. Emotional Development and Emotional Intelligence: Implications for Educators.

[24] Domínguez-García E., Fernández-Berrocal P. (2018). The Association Between Emotional Intelligence and Suicidal Behavior: A Systematic Review. Front. Psychol..

[25] Mikolajczak M., Roy E., Luminet O., Fillée C., de Timary P. (2007). The moderating impact of emotional intelligence on free cortisol responses to stress. Psychoneuroendocrinology.

[26] Salovey P., Stroud L.R., Woolery A., Epel E.S. (2002). Perceived Emotional Intelligence, Stress Reactivity, and Symptom Reports: Further Explorations Using the Trait Meta-Mood Scale. Psychol. Health.

[27] Berrios M.P., Extremera N., Nieto-Flores M.P. (2016). Exploring the socio-emotional factors associated with subjective well-being in the unemployed. PeerJ.

[28] Knopp K.A. (2016). Exploring the relationship of emotional intelligence with mental health status in polish unemployed persons–differences between men and women. Polish Psychol. Bull..

[29] Hodzic S., Ripoll P., Bernal C., Zenasni F. (2015). The Effects of Emotional Competences Training among Unemployed Adults: A Longitudinal Study. Appl. Psychol. Heal. Well-Being.

[30] O’Connell P.J., Mcguinness S., Kelly E. (2012). The transition from short- to long-term unemployment: A statistical profiling model for Ireland. Econ. Soc. Rev..

[31] Maier R., Egger A., Barth A., Winker R., Osterode W., Kundi M., Wolf C., Ruediger H. (2006). Effects of short- and long-term unemployment on physical work capacity and on serum cortisol. Int. Arch. Occup. Environ. Health.

[32] Hendricks V.M., Blanken P., Hendricks V.M., Blanken P., Adriaans N. (1992). Snowball sampling: Theoretical and practical considerations. Snowball Sampling: A Pilot Study on Cocaine Use.

[33] Blau G., Petrucci T., McClendon J. (2013). Correlates of life satisfaction and unemployment stigma and the impact of length of unemployment on a unique unemployed sample. Career Dev. Int..

[34] Extremera N., Rey L. (2014). Health-related quality of life and cognitive emotion regulation strategies in the unemployed: a cross-sectional survey. Health Qual. Life Outcomes.

[35] Mérida-López S., Extremera N., Rey L. (2018). Understanding the Links Between Self-Report Emotional Intelligence and Suicide Risk: Does Psychological Distress Mediate This Relationship Across Time and Samples?. Front. Psychiatry.

[36] Extremera N., Rey L., Sánchez-Álvarez N. (2019). Validation of the Spanish version of Wong Law Emotional Intelligence Scale (WLEIS-S). Psicothema.

[37] Law K.S., Wong C.-S., Song L.J. (2004). The construct and criterion validity of emotional intelligence and its potential utility for management studies. J. Appl. Psychol..

[38] Cohen S., Kamarck T., Mermelstein R. (1983). A Global Measure of Perceived Stress. J. Health Soc. Behav..

[39] Remor E., Carrobles J. (2001). Versión Española de la Escala de Estrés Percibido (PSS-14): Estudio psicométrico en una muestra VIH+. Ansiedad y Estrés.

[40] Extremera N., Rey L. (2015). The moderator role of emotion regulation ability in the link between stress and well-being. Front. Psychol..

[41] Vilagut G., Valderas J.M., Ferrer M., Garin O., López-García E., Alonso J. (2008). Interpretación de los cuestionarios de salud SF-36 y SF-12 en España: Componentes físico y mental. Med. Clin..

[42] Ware J.E., Kosinski M., Keller S.D. (1996). A 12-Item Short-Form Health Survey. Med. Care.

[43] Osman A., Bagge C.L., Gutierrez P.M., Konick L.C., Kopper B.A., Barrios F.X. (2001). The Suicidal Behaviors Questionnaire-Revised (SBQ-R):Validation with Clinical and Nonclinical Samples. Assessment.

[44] Hayes A. (2013). Introduction to Mediation, Moderation, and Conditional Process Analysis: A Regression-based Approach.

[45] Overholser J.C., Braden A., Dieter L. (2012). Understanding suicide risk: Identification of high-risk groups during high-risk times. J. Clin. Psychol..

[46] Hayes A.F. (2015). An Index and Test of Linear Moderated Mediation. Multivariate Behav. Res..

[47] Mikolajczak M., Avalosse H., Vancorenland S., Verniest R., Callens M., van Broeck N., Fantini-Hauwel C., Mierop A. (2015). A nationally representative study of emotional competence and health. Emotion.

[48] Lee E.H. (2012). Review of the psychometric evidence of the perceived stress scale. Asian Nurs. Res..

[49] Goldman S.L., Kraemer D.T., Salovey P. (1996). Beliefs about mood moderate the relationship of stress to illness and symptom reporting. J. Psychosom. Res..

